# Software tool for visualization of a probabilistic map of the epileptogenic zone from seizure semiologies

**DOI:** 10.3389/fninf.2022.990859

**Published:** 2022-10-13

**Authors:** Fernando Pérez-García, Ali Alim-Marvasti, Gloria Romagnoli, Matthew J. Clarkson, Rachel Sparks, John S. Duncan, Sébastien Ourselin

**Affiliations:** ^1^Department of Medical Physics and Biomedical Engineering, University College London, London, United Kingdom; ^2^Wellcome/EPSRC Centre for Interventional and Surgical Sciences (WEISS), University College London, London, United Kingdom; ^3^School of Biomedical Engineering & Imaging Sciences (BMEIS), King's College London, London, United Kingdom; ^4^Department of Clinical and Experimental Epilepsy, UCL Queen Square Institute of Neurology, University College London, London, United Kingdom; ^5^National Hospital for Neurology and Neurosurgery, London, United Kingdom

**Keywords:** epilepsy surgery, visualization, semiology, presurgical evaluation, epileptogenic zone, electroencephalography

## Abstract

Around one third of epilepsies are drug-resistant. For these patients, seizures may be reduced or cured by surgically removing the epileptogenic zone (EZ), which is the portion of the brain giving rise to seizures. If noninvasive data are not sufficiently lateralizing or localizing, the EZ may need to be localized by precise implantation of intracranial electroencephalography (iEEG) electrodes. The choice of iEEG targets is influenced by clinicians' experience and personal knowledge of the literature, which leads to substantial variations in implantation strategies across different epilepsy centers. The clinical diagnostic pathway for surgical planning could be supported and standardized by an objective tool to suggest EZ locations, based on the outcomes of retrospective clinical cases reported in the literature. We present an open-source software tool that presents clinicians with an intuitive and data-driven visualization to infer the location of the symptomatogenic zone, that may overlap with the EZ. The likely EZ is represented as a probabilistic map overlaid on the patient's images, given a list of seizure semiologies observed in that specific patient. We demonstrate a case study on retrospective data from a patient treated in our unit, who underwent resective epilepsy surgery and achieved 1-year seizure freedom after surgery. The resected brain structures identified as EZ location overlapped with the regions highlighted by our tool, demonstrating its potential utility.

## 1. Introduction

Anti-seizure medication (ASM) is the first-line treatment for epilepsy. However, one third of epilepsies are drug-resistant (Engel, 2016). The epileptogenic zone (EZ) is “the area of cortex indispensable for the generation of clinical seizures” (Rosenow and Lüders, 2001). If the EZ can be localized precisely, curative resective surgery may be performed. However, only 40–70% of patients remain seizure-free after surgery (Jobst and Cascino, 2015). The location of the EZ is normally inferred by a multidisciplanary team, following a multimodal non-invasive evaluation including different tools such as magnetic resonance image (MRI), neuropsychological tests, and seizure semiology reported by patients and witnesses, and recorded by video-electroencephalography (EEG). If the information regarding the location of the EZ is clear and concordant between the different evaluation tests, curative resection may be performed to treat the epilepsy. Otherwise intracranial EEG (iEEG) electrodes may be implanted to localize the EZ precisely. To determine the brain regions that need to be implanted with electrodes, i.e., the targets, the team leverages information from the previously described non-invasive examinations. The choice of targets is therefore influenced by the team's subjective experience and personal knowledge of the literature. This leads to substantial variations of implantation strategies across different epilepsy centers (Tufenkjian and Lüders, 2012). Whilst the symptomatogenic and epileptogenic zones may not be coterminous, if a patient with particular semiological features becomes seizure-free after resection of a certain brain region, it is a reasonable inference considering that semiological feature as a marker for the curative resection of that area. The diagnostic pathway for surgical planning could therefore be supported and standardized by an objective tool to aid clinicians in deducing the possible EZ location from patient seizure semiology.

It would be useful for clinicians to be able to quickly and easily assess relevant studies in the literature for a specific semiology. Such a method has been proposed using a large number of studies analyzed *via* the Preferred Reporting Items for Systematic Reviews and Meta-Analyzes (PRISMA) framework (Page et al., 2021) to generate *Semio2Brain*, a database of entries mapping seizure semiologies to brain regions (Alim-Marvasti et al., 2022b). Clinicians and researchers would also benefit from a easy-to-use and intuitive graphical user interface (GUI) to such a database. Moreover, visualizing the probability of each structure containing the EZ on 3D multimodality imaging (3DMMI) could help plan resection or iEEG implantation strategies (Nowell et al., 2015, 2017), and potentially be used to guide automatic iEEG trajectory planning (ATP) (Sparks et al., 2017). Finally, a 3DMMI visualization could be used to perform qualitative and quantitative analyzes of information contained in the database and further refine its utility to predict the EZ.

In this work, we present the Semiology Visualization Tool (SVT), a software tool that, given an observed list of seizure semiologies, provided by clinical observation, and other patient data, such as the dominant hemisphere, displays probable EZ locations in an intuitive way. The SVT provides a table with the number of datapoints associated with each brain region, where each datapoint represents a patient reported in the literature presenting the observed semiology, with an EZ objectively determined to arise from the brain region, e.g., becoming seizure-free after resection of that region (Table 1). Then, the information in the table is additionally represented using an intuitive 3DMMI visualization, personalized to each patient's imaging data, where locations that are likely to be in the EZ are highlighted with brighter colors (**Figure 5**).

**Table 1:** Result of querying an imaginary database with the semiology term *Head version* and exemplar brain structures A, B, and C, assuming that the brain has been parcellated into only three structures.

	**Structure A**	**Structure B**	**Structure C**
Head version	0	5	20

In this example, according to the hypothetical literature analyzed to build the database, structure C was one of the resected structures in 20 patients (datapoints) presenting *Head version* who became seizure-free after surgery, suggesting a high probability of the EZ being associated with that structure. Structure B was resected for five patients who became seizure-free after surgery. Structure A was never resected in patients who presented *Head version* and became seizure-free after surgery. This result would support the decision of implanting electrodes in structure C (and possibly B), as they are likely to be associated with the EZ according to the retrospective information in the literature.

## 2. Methods

In this section, we describe the design and workflow of the SVT, including implementation details and dependencies.

**Figure 1:**
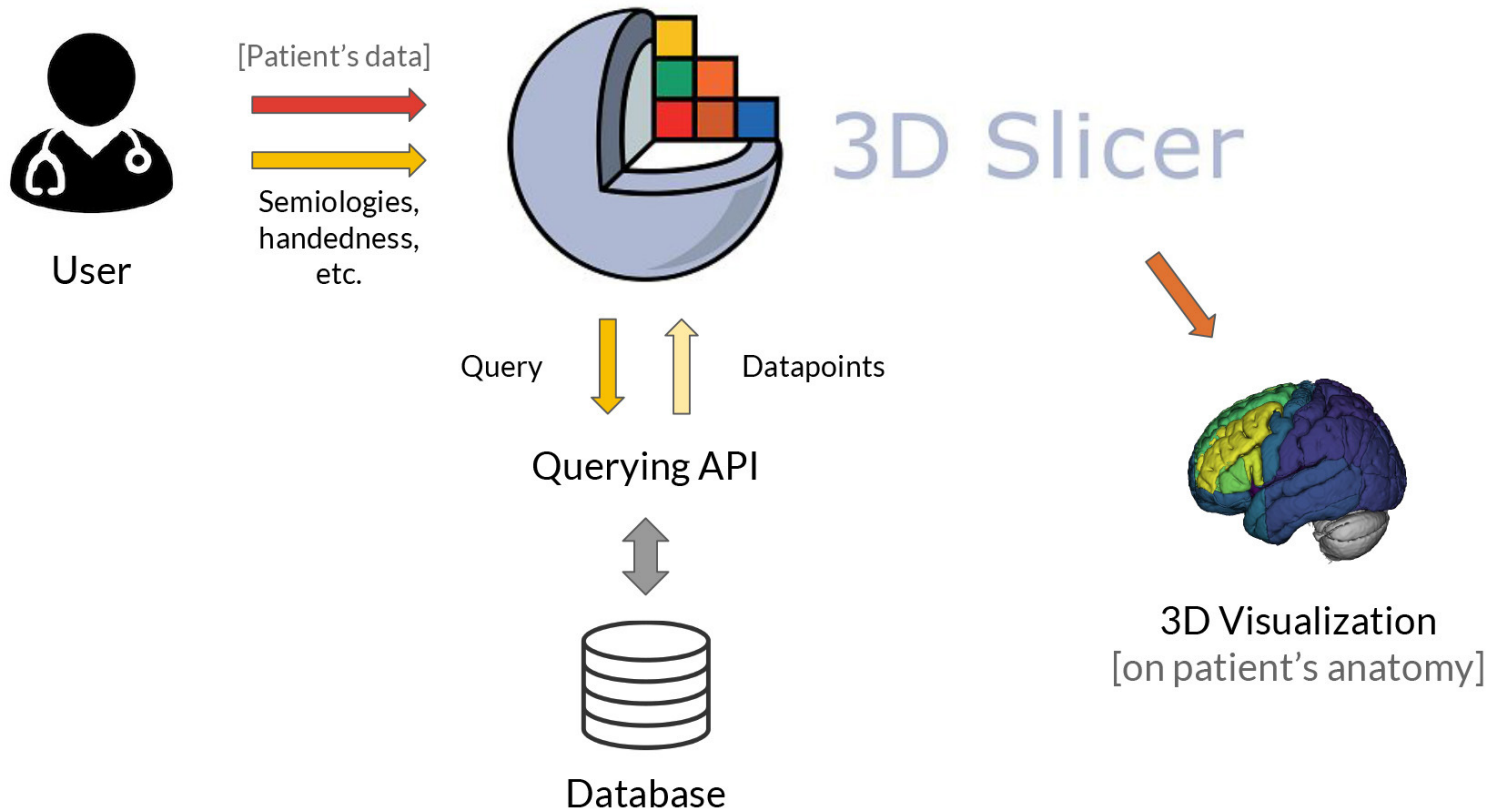
General architecture of the SVT. 1) The user inputs a list of observed seizure semiologies and (optionally) patient's data comprising an MRI and brain parcellation of the patient for visualization in the patient's space. 2) The GUI of the 3D Slicer module is used to input the observed semiologies to the software, without the need to code. 3) The 3D Slicer module generates a structured query that is sent to be processed by the querying API to access the database. 4) The database is queried and the retrieved datapoints are converted into a 3DMMI visualization showing the EZ probability map overlaid to the patient's anatomy, if provided.

**Figure 2:**
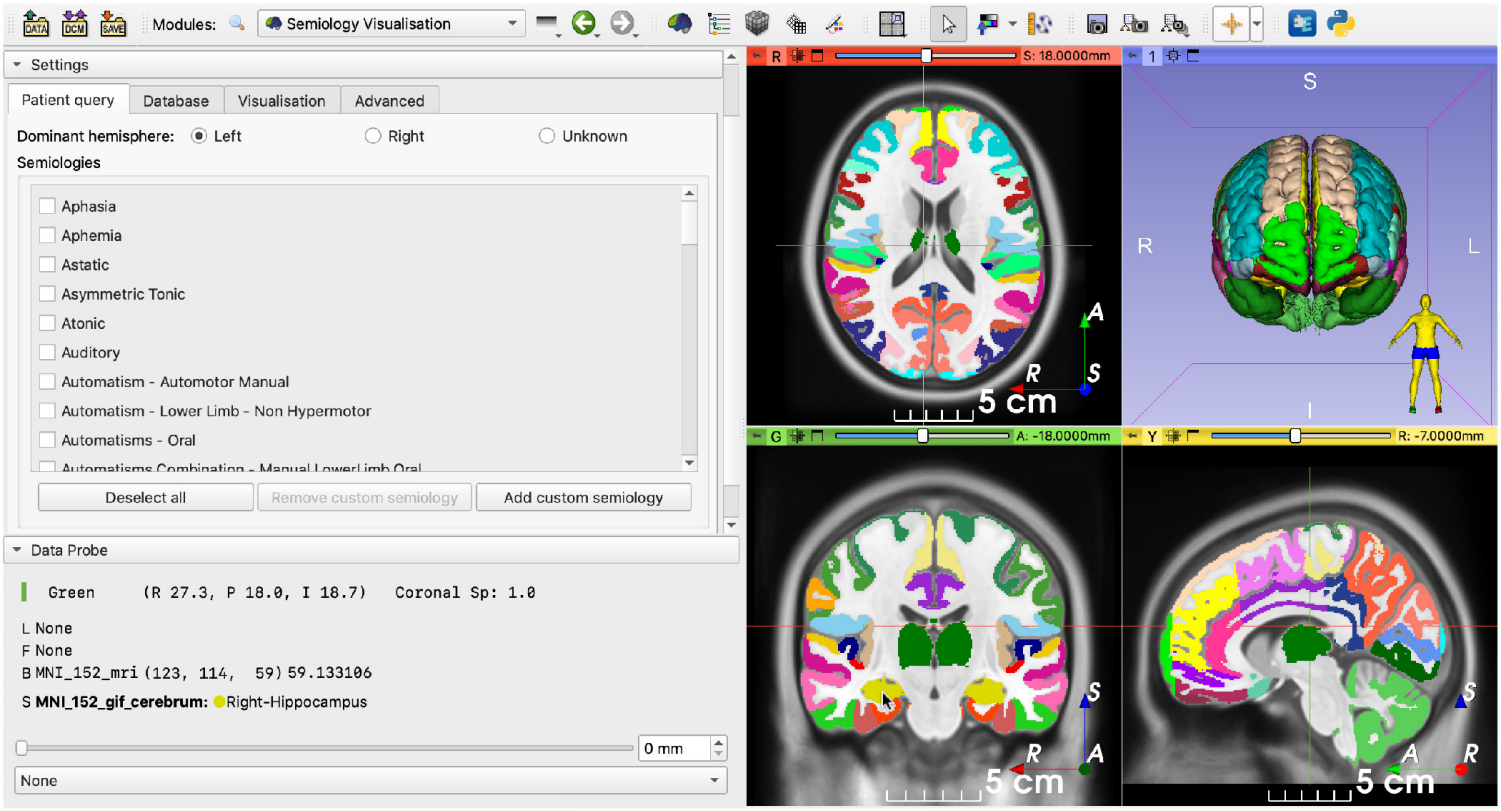
Semiology visualization module after loading an MNI template as image reference and its corresponding brain parcellation. **(Left)** Main panel containing a list of suggested semiology terms to be selected by the user; **(Right)** Images in the patient's space. Images are shown using the radiological convention, i.e., the left hemisphere is shown on the right and vice versa.

As a general overview (Figure 1), the user is first prompted to load a patient's MRI that will be used as reference image and a corresponding brain parcellation that must be compatible with the software package used to query the database (Section 2.3). Then, a list of pre-defined semiology terms and additional settings compatible with the application programming interface (API) are displayed, and the user fills in the observed semiologies (Figure 2 and Section 2.4). Finally, the query is submitted using the API, which returns the table of datapoints that is used to generate the 3D EZ probability map (**Figures 5**, **7** and Sections 2.5, 2.6). Each step takes only a few seconds, making the whole process normally 1 or 2 min long.

### 2.1. Implementation

The SVT is a 3D Slicer Python module (Fedorov et al., 2012). 3D Slicer is “a free, open source and multi-platform software package widely used for medical, biomedical, and related imaging research” ^1^. As with 3D Slicer, the SVT can be used on all major platforms: Windows, Linux and macOS.

The SVT leverages the libraries on which 3D Slicer is built: the Insight Toolkit (ITK) for image processing (McCormick et al., 2014), the Visualization Toolkit (VTK) for visualization (Schroeder et al., 2006) and Qt for the cross-platform GUI ^2^.

The Python code for the SVT is available on GitHub^3^. We have also developed a light-weight online version hosted on Binder (Bussonnier et al., 2018), which does not require installation of 3D Slicer and can be accessed through a web browser^4^.

### 2.2. Dependencies

The SVT depends on an external API that must be able to provide the following:

1. At start-up, a list of pre-defined semiologies that are used to generate the GUI and a list of additional settings to perform the database query.
2. After the database is queried with a list of semiologies and additional settings, a table with datapoints associated with each brain structure.

The usage examples in this paper use the *Semio2Brain* database and the corresponding developed software to query the database (Alim-Marvasti et al., 2022a,b), which were defined according to the Neuromorphometrics atlas parcellation scheme^5^.

To query the *Semio2Brain* database, in this work we used the mega_analysis package, which can be installed using Pip Installs Packages (PIP) (Alim-Marvasti et al., 2022a). The mega_analysis software package is installed within the 3D Slicer Python environment in the background, when loading the module, unless it was already installed.

### 2.3. Data loading

The user is asked to load the patient's MRI and a corresponding brain parcellation. The default behavior when the user opts not to load imaging data, or the SVT is being used to explore the database and not for surgical planning, is to load a generic Montreal Neurological Institute (MNI) template (Fonov et al., 2009). As the EZ is expected to be in the gray matter, the brain parcellation is automatically stripped of white matter and other irrelevant structures such as cerebrospinal fluid (CSF), brainstem and cerebellum, for visualization and reporting purposes. The input brain parcellation must be chosen based on the parcellation scheme in the database being queried. In this work, we use the *Semio2Brain* database, which uses the Neuromorphometics atlas integrated in the geodesical information flows (GIF) parcellation tool (Cardoso et al., 2015). However, the SVT is agnostic to the atlas choice.

Finally, 3D meshes are generated for visualization using the marching cubes algorithm (Lorensen and Cline, 1987; Pinter et al., 2019). The user is then presented with the preprocessed data (Figure 2).

### 2.4. Input semiologies

**Figure 3:**
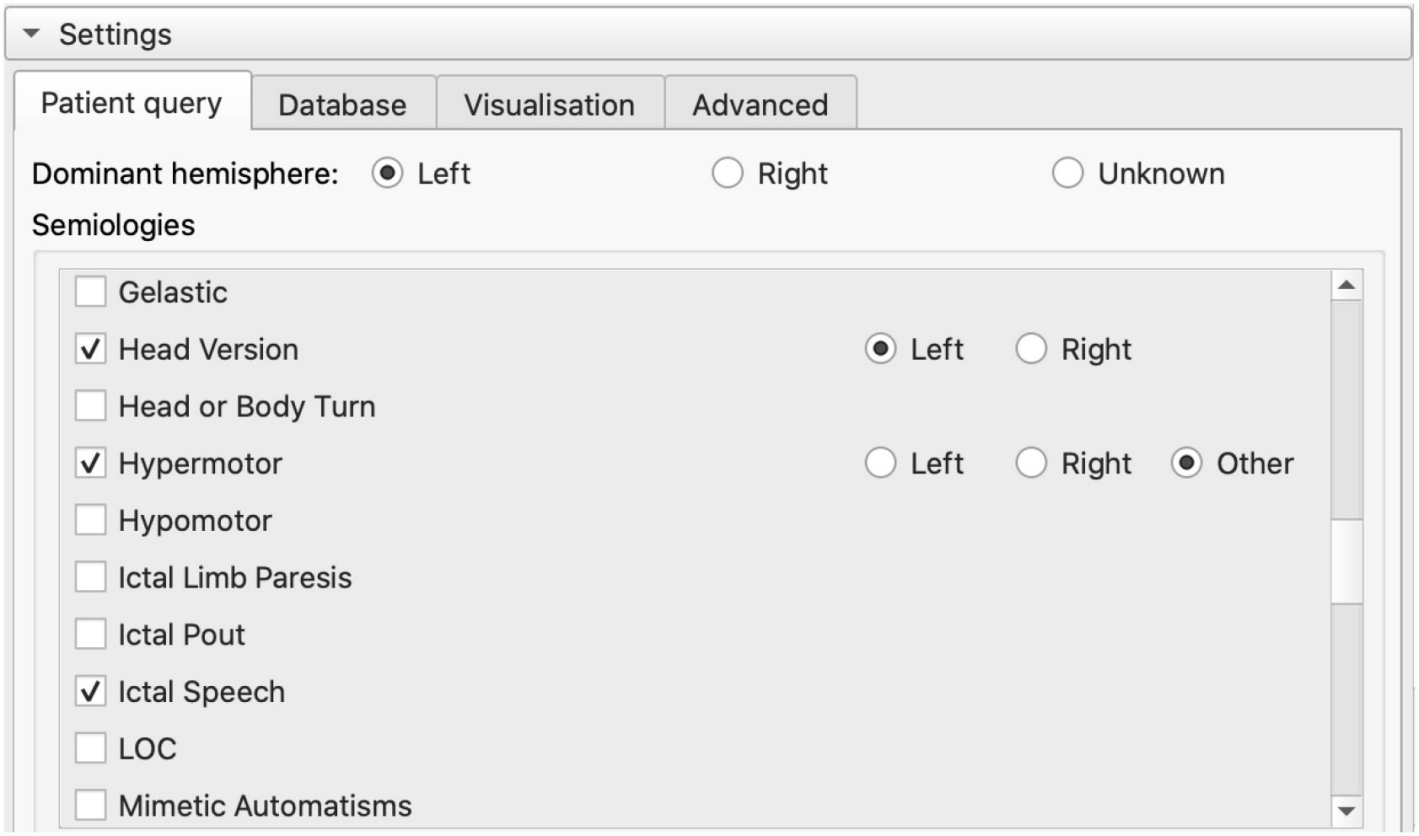
List of pre-defined semiologies in the GUI. Users can select a semiology in the list or add a custom one. Custom semiologies are matched to a pre-defined semiology using regular expressions to map concepts associated with that semiology.

Once the image data have been loaded, the pre-defined list of common semiologies is shown on the left-hand side of the screen (Figures 2, 3). Certain semiologies such as *Head version* require a laterality (left or right). Semiologies such as *Hypermotor* are allowed to have no associated laterality, indicating it was observed for both sides. Some semiologies such as *Ictal speech* never have an associated laterality. The dominant hemisphere may also be specified if known, helping to narrow results.

**Figure 4:**
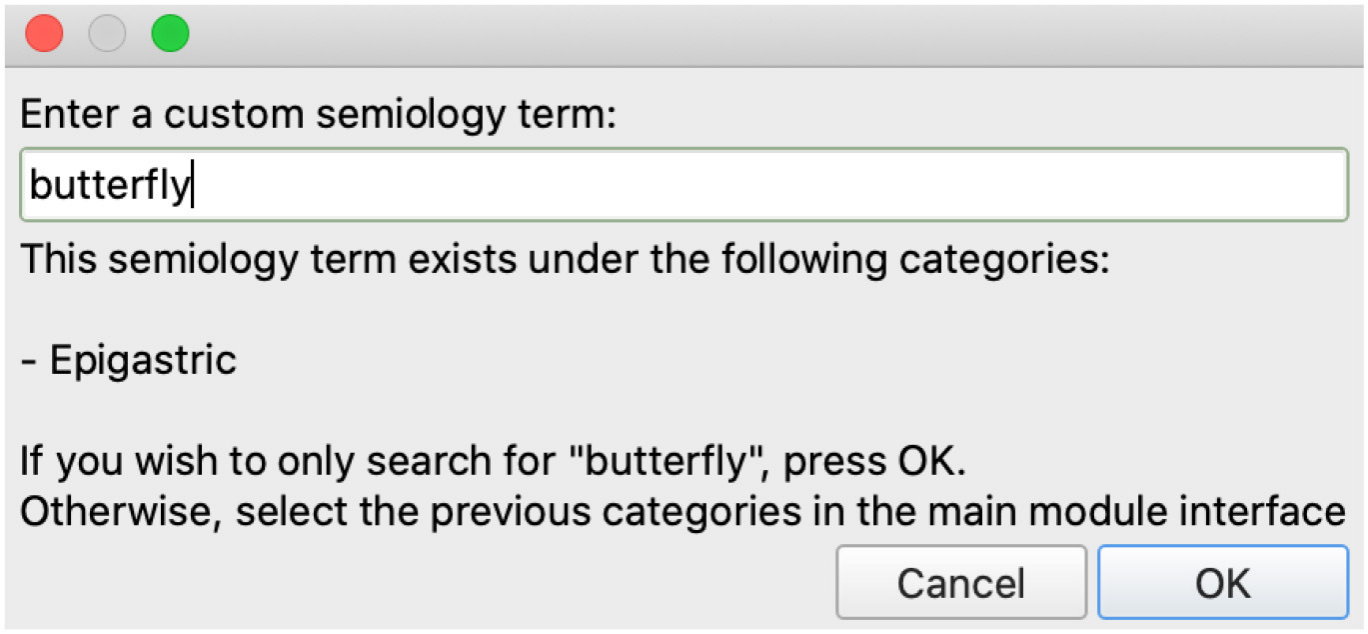
Adding a custom semiology term to the semiology visualization module. For example, ‘butterflies' and ‘déjà vu' would be matched with epigastric and psychic auras, respectively. In this case, the term *butterfly* was entered as the patient described “a butterfly feeling in my stomach”. The module used to query the database matches the input term with the *Epigastric* semiology term, and therefore the 3D Slicer module suggests matching *Epigastric* to the list of selected semiologies.

Additionally, the mega_analysis querying API allows for custom semiologies to be entered, which may be matched to one of the pre-defined semiologies using regular expressions (Alim-Marvasti et al., 2022a). Matching is performed in real time within the GUI whenever the characters in the widget are modified, as long as three or more characters have been entered (Figure 4).

### 2.5. Querying the database

The SVT reads the user specified semiologies and settings from the GUI and generates a machine-readable data structure to query the database. As the waiting time to query the database is sometimes in the order of tens of seconds, previous query results are cached in the disk for faster retrieval using YAML Ain't Markup Language (YAML), a human-readable file format. The result from querying the database is a table containing the number of datapoints for each brain structure (Table 1). Each datapoint represents a patient case from the literature presenting the observed semiology, where the EZ was objectively demonstrated to be related to the brain region after observing seizure freedom post-resection. If multiple semiologies are selected, results are combined and a number between 0 and 1 is computed for each brain structure by the database query package (in this work, mega_analysis). Details on how the datapoints for multiple semiologies are combined can be found in Alim-Marvasti et al. (2022a).

### 2.6. Visualization

**Figure 5:**
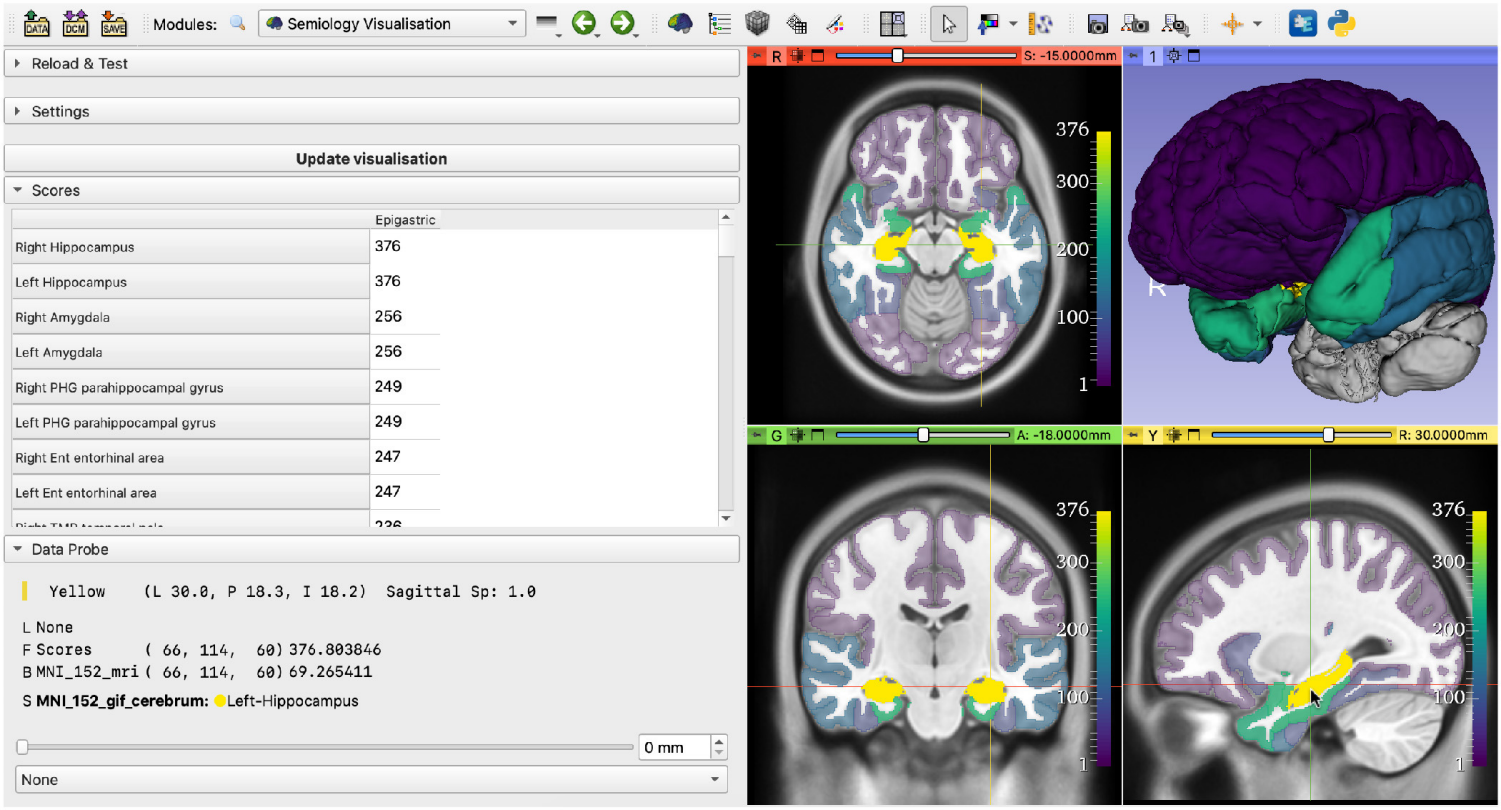
Results of querying the database with the *Epigastric* semiology. **(Left)** Table showing the number of datapoints associated with each brain structure; **(Right)** EZ probability map, where brightness (and opacity, on the 2D views) is linearly proportional to the number of datapoints.

Results from the database query are shown to the user in two ways: a table and a rendering leveraging the brain parcellation image loaded by the user. The datapoints table is displayed on the GUI and the list of structures is sorted by the number of datapoints, showing first the structures with the highest number of datapoints in the database (Figures 5, **7**).

**Figure 6:**
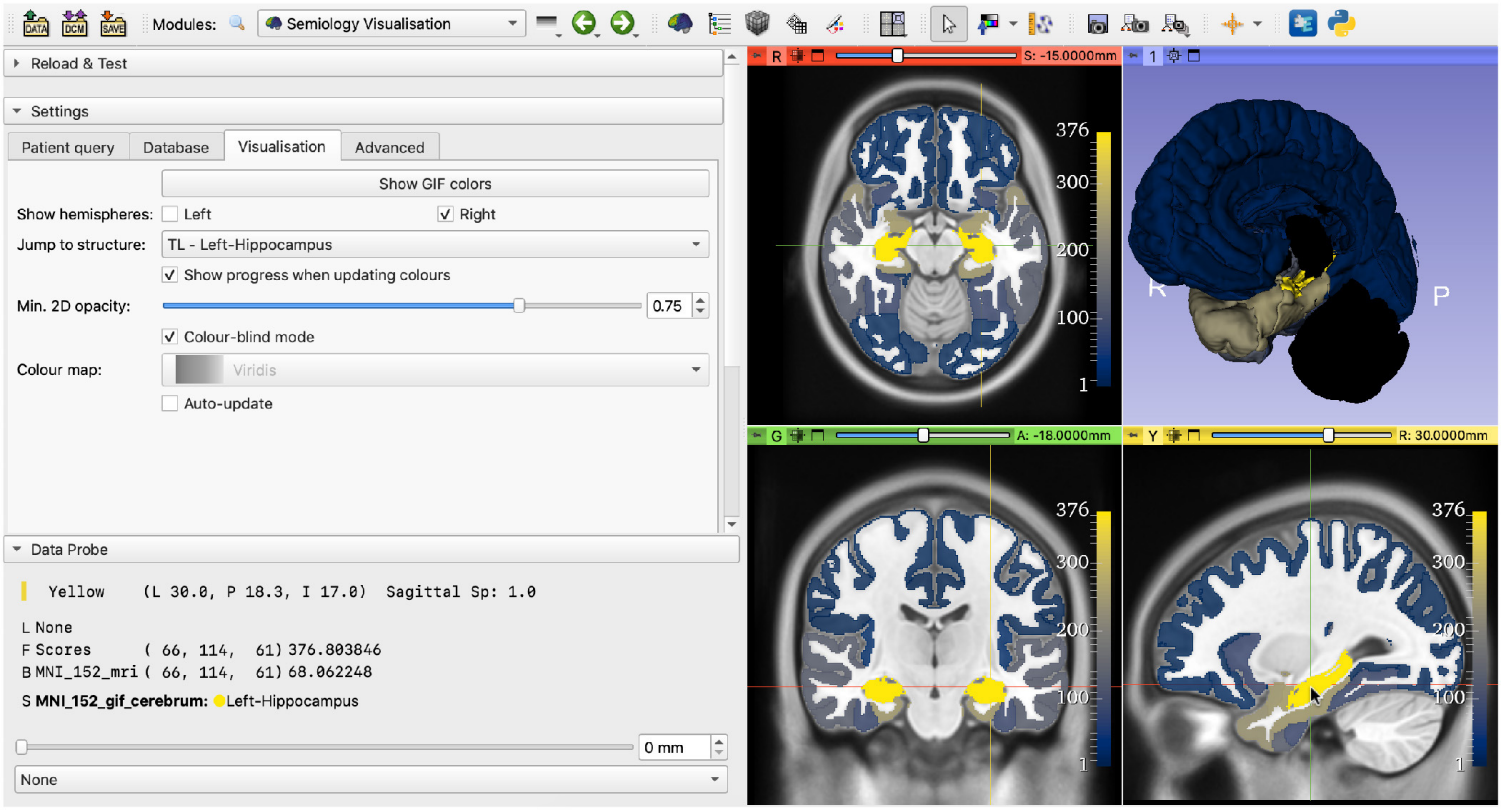
Demonstration of advanced visualization settings. The selected settings are: show only the right hemisphere, center the views on the right thalamus, set minimum opacity to 75% (default is 25%) and enable color-blind mode.

**Figure 7:**
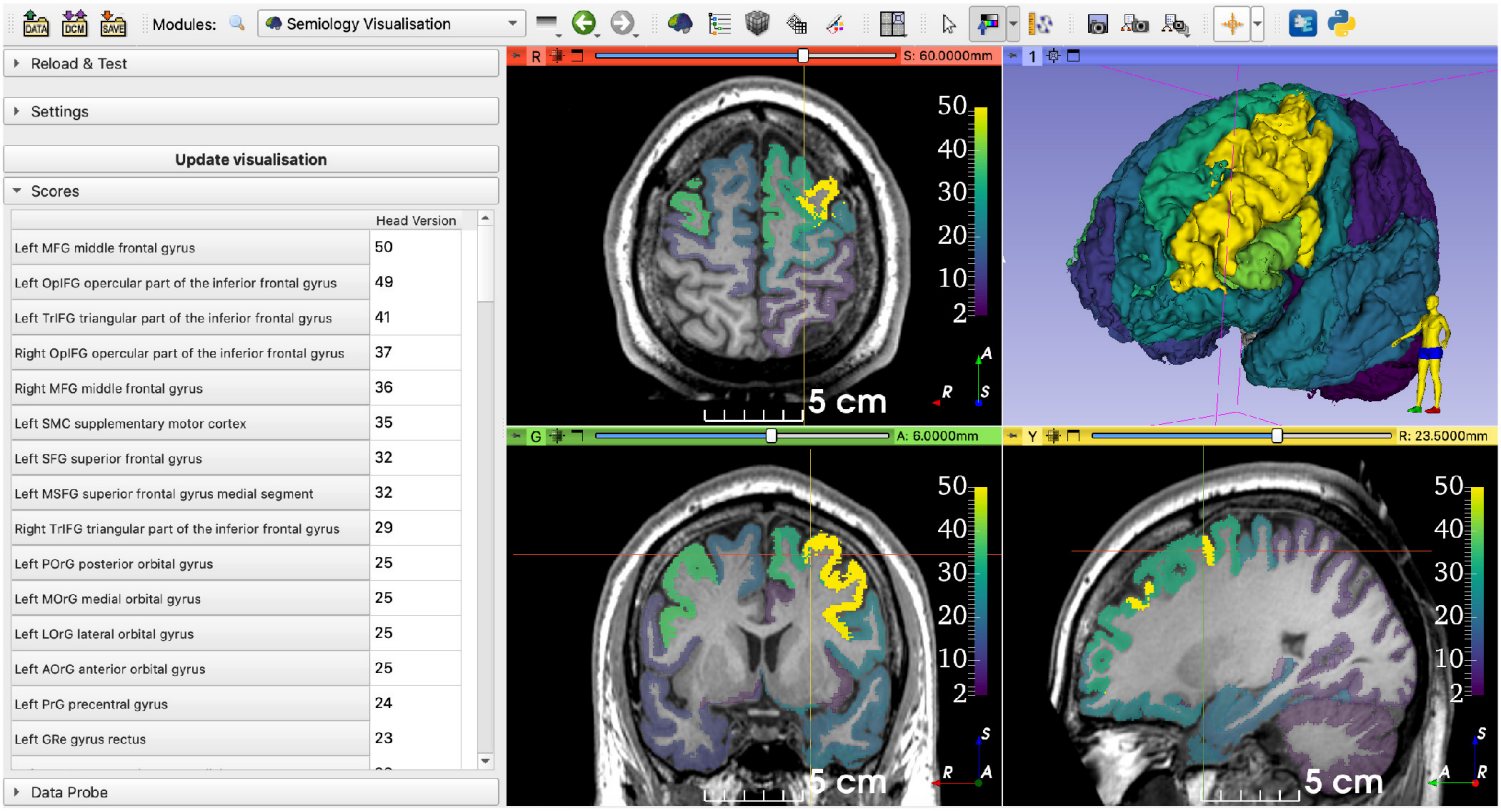
Querying the database using data from a retrospective patient case. The selected semiology term was *Head version (right)*, and the dominant hemisphere was *Left*. The regions with highest numbers of datapoints concentrate around the left frontal lobe (represented on the right side of the axial and coronal views).

A probability map showing the datapoints per brain parcellation label is generated and displayed on the 2D slice views and the 3D view. The 2D slice views are centered on the brain structure with the highest number of associated datapoints. Brain structures without datapoints are hidden from the 2D slice views and shown in gray on the 3D view. To emphasize the importance of brain structures with a high number of datapoints, the opacity of each structure on the 2D slice views is adjusted to be linearly proportional to the number of associated datapoints. We chose the open-source, perceptually uniform, sequential colormap *viridis* as default for this application. This implies that the brightness of each structure is also linearly proportional to the number of associated datapoints. Other colormaps are available as a user-selectable option, if desired. The colorbars on the 2D slice views help create a mental mapping from colors to number of datapoints (Figures 5–**7**).

Advanced visualization settings may be selected by the users. Settings include showing only one hemisphere on the 3D view, setting the minimum opacity on the 2D views or enabling color blind mode, in which the color-blind-friendly *cividis* colormap is used (Nuñez et al., 2018) (Figure 6).

## 3. Results

In this section, we demonstrate a case study example of our SVT for a retrospective analysis of a patient with epilepsy who underwent resective surgery at the National Hospital for Neurology and Neurosurgery (NHNN) (Queen Square, London, UK). For all analyzes, we use the mega_analysis querying module, which uses the *Semio2Brain* database (Alim-Marvasti et al., 2022a,b).

The patient was right-handed and presented head version to the right at the beginning of seizures. Ictal EEG was nonlateralizing and interictal EEG showed bitemporal sharp waves. We used the patient's preoperative *T*_1_-weighted MRI as reference for the visualization. The Neuromorphometrics brain parcellation was generated using GIF (Cardoso et al., 2015).

We queried the *Semio2Brain* using the semiology *Head version (right)* and setting the dominant hemisphere to *Left*. The most highlighted structures concentrate in the left frontal lobe (Figure 7).

**Figure 8:**
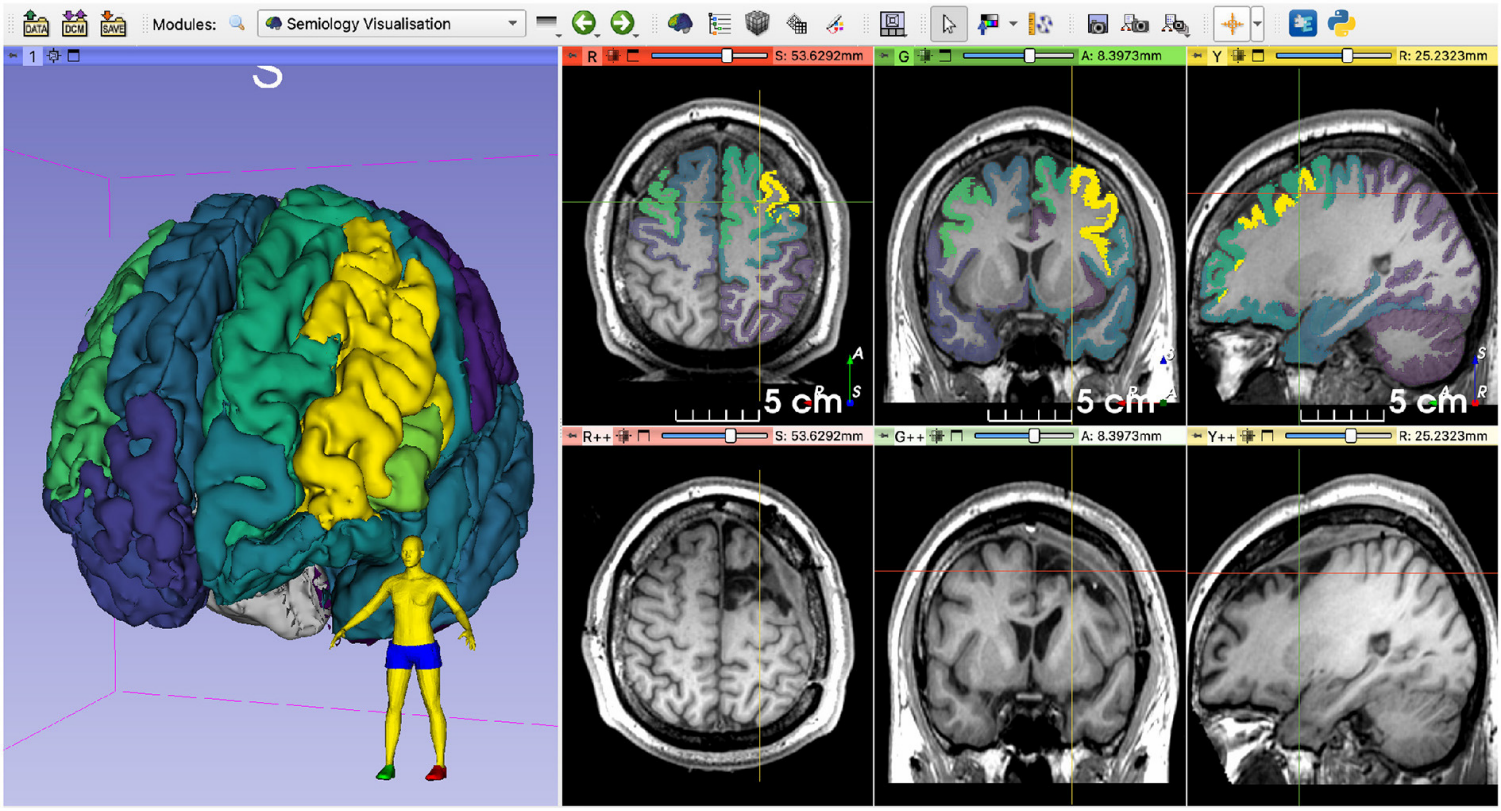
Qualitative comparison of the EZ probability map and the postoperative MRI. Note the overlap between the highlighted structures and the resection cavity. The images were rigidly registered, and the crosshairs are centered on approximately the same region. The patient became seizure-free after resective surgery.

We used a rigid registration algorithm to align the preoperative and postoperative MRIs for visualization purposes (Ourselin et al., 2000). As non-rigid brain deformations may happen after surgery and we used a rigid registration, the alignment is only approximate; however, it has sufficient accuracy to enable a visual analysis. When visualizing the aligned images, including the probability map, we observed an overlap between the highlighted areas (i.e., the brain structures with the highest number of datapoints) and the resection cavity (Figure 8). As this is a retrospective case from a patient who underwent resective surgery several years ago, the postoperative MRI and the clinical outcome are available. The surgery was successful and resulted in complete seizure during a 4-year follow-up.

## 4. Discussion

The choice of targets for iEEG electrodes implantation would benefit from an objective, data-driven method. In this work, we present an open-source software tool to visualize regions of the brain with a high probability of being associated with the EZ, given a set of observed seizure semiologies. To the best of our knowledge, our tool is the first one to provide evidence-based, quantitative and qualitative insights on the probable EZ locations using 3DMMI.

The SVT is not designed to replace clinicians in planning the iEEG implantation or the resective surgery, but to support their decisions using a data-driven approach that displays patterns in the literature intuitively and objectively. Our expectation is that the results of this analysis may increase the number of cerebral areas targeted with iEEG, but would not reduce them. This could be caused by the SVT highlighting regions not previously considered due to the experience of the team but which the literature suggests may be implicated.

In the future, we will improve the generalizability of our framework to improve the compatibility with custom databases and other brain parcellation strategies, such as the Desikan-Killiany atlas (Desikan et al., 2006). Additional development of a full online version of our SVT hosted on a well-established cloud infrastructure service, such as Microsoft Azure or Google Cloud would allow for a fast and seamless user experience. Another potential improvement is adding support to parcellate the brain at loading time using a deep learning model (Li et al., 2017; Pérez-García, 2019), that satisfies the requirements of the selected database and corresponding API. This would spare the user the need to wait for hours before the parcellation is generated, which is typically the case for GIF (Cardoso et al., 2015) or FreeSurfer^6^.

## Data availability statement

The datasets presented in this study can be found in online repositories. The names of the repository/repositories and accession number(s) can be found below: https://github.com/fepegar/Semiology-Visualisation-Tool/archive/refs/tags/v1-paper.zip.

## Author contributions

FP-G wrote the first draft of the manuscript. FP-G and AA-M contributed to the software development. All authors contributed to conception and design of the study and manuscript revision, read, and approved the submitted version.

## Funding

This work was supported by the Engineering and Physical Sciences Research Council (EPSRC) [EP/R512400/1]. This work was additionally supported by the EPSRC-funded UCL Centre for Doctoral Training in Intelligent, Integrated Imaging in Healthcare (i4health) [EP/S021930/1] and the Wellcome/EPSRC Centre for Interventional and Surgical Sciences (WEISS, UCL) [203145Z/16/Z]. This publication represents, in part, independent research commissioned by the Wellcome Innovator Award [218380/Z/19/Z].

## Conflict of interest

The authors declare that the research was conducted in the absence of any commercial or financial relationships that could be construed as a potential conflict of interest.

## Publisher's note

All claims expressed in this article are solely those of the authors and do not necessarily represent those of their affiliated organizations, or those of the publisher, the editors and the reviewers. Any product that may be evaluated in this article, or claim that may be made by its manufacturer, is not guaranteed or endorsed by the publisher.

## Author disclaimer

The views expressed in this publication are those of the authors and not necessarily those of the Wellcome Trust.

## Footnotes

^1^ https://www.slicer.org/

^2^ https://www.qt.io

^3^ https://github.com/fepegar/Semiology-Visualisation-Tool/archive/refs/tags/v1-paper.zip

^4^ https://mybinder.org/v2/gh/fepegar/SVT-web/HEAD?filepath=SVT-web.ipynb

^5^ http://www.neuromorphometrics.com

^6^ https://surfer.nmr.mgh.harvard.edu/
